# Expression of Calretinin, Marker of Mesothelial Differentiation, in Pancreatic Ductal Adenocarcinoma: A Potential Diagnostic Pitfall

**DOI:** 10.5146/tjpath.2020.01519

**Published:** 2021-05-15

**Authors:** Gokce Askan, Olca Basturk

**Affiliations:** Department of Pathology, Rize University Training and Research Hospital, Rize, Turkey; Memorial Sloan Kettering Cancer Center, New York, USA

**Keywords:** Calretinin, Pancreatic ductal adenocarcinoma, Poorly differentiated, Undifferentiated, Mesothelioma

## Abstract

***Objective:*** Pancreatic ductal adenocarcinoma is one of the most common causes of “peritoneal carcinomatosis” and has an insidious growth pattern. Thus, it falls into the differential diagnosis of other peritoneal malignancies including malignant mesothelioma. Recently, we have encountered an undifferentiated pancreatic carcinoma presenting with peritoneal disease and exhibiting immunoreactivity to calretinin, mimicking mesothelioma. In this study, we explored the incidence of calretinin expression in pancreatic ductal adenocarcinoma.

***Materials and Methods: *** Calretinin immunohistochemical staining was performed on the tissue microarrays (TMAs), which were created using three 0.6 mm diameter punches per tumor (n=113). Distribution and intensity of expression were evaluated.

***Results: *** The TMAs contained 86 well/moderately differentiated and 27 poorly differentiated/undifferentiated carcinomas. Calretinin was positive in nine tumors (8%); six with diffuse and strong staining, three with focal and/or weak staining. The incidence of calretinin expression was 15% in poorly differentiated/undifferentiated carcinomas (vs. 6% in well/moderately differentiated carcinomas, p=0.03).

***Conclusions: *** Pancreatic ductal adenocarcinomas, especially when poorly differentiated/undifferentiated, may be diffusely and strongly positive for calretinin creating a potential diagnostic challenge with malignant mesothelioma. Therefore, caution should be exercised when using this marker to explore a diagnosis of malignant mesothelioma. Tumors expressing calretinin without other mesothelial markers should prompt a careful evaluation of the morphologic and immunohistochemical features to exclude other malignancies. If the diagnosis of pancreatic ductal adenocarcinoma is considered, ductal differentiation can be demonstrated by using additional immunohistochemical markers such as mucin-related glycoproteins (MUC1, MUC5AC) and/or oncoproteins (CEA, B72.3, CA125).

## INTRODUCTION

Pancreatic ductal adenocarcinoma (PDAC) is one of the most common causes of “peritoneal carcinomatosis” and has an insidious growth pattern (1–8). Thus, when it is poorly differentiated, it falls into the differential diagnosis of other peritoneal malignancies including malignant mesothelioma. However, distinguishing metastatic adenocarcinomas from malignant mesotheliomas, especially of the epithelial subtype, is difficult on purely morphological grounds. Therefore, additional work-up (a panel of immunohistochemical stains) is performed to establish the diagnosis.

Calretinin is a calcium binding protein, structurally related to S100 and inhibin, commonly expressed in a wide variety of normal cells including mesothelial cells as well as in certain neoplasms such as malignant mesothelioma (9–13). In fact, in daily practice, it is regarded as one of the most sensitive immunohistochemical markers for malignant mesothelioma (11,13–16).

However, we have recently encountered an undifferentiated carcinoma of the pancreas presenting with peritoneal disease and exhibiting immunoreactivity to calretinin, mimicking malignant mesothelioma, not only morphologically but also immunohistochemically. Since the literature on calretinin expression in PDAC is very limited and mainly based on a few cases buried in a series of adenocarcinomas from various organs, we explored the incidence of calretinin expression in a large series of PDACs in this study (11).

## MATERIAL and METHODS

With approval of the Institutional Review Board (Date: 12/26/2019, Protocol # 16-1683), 113 PDACs were retrieved from the files of the Department of Pathology at Memorial Sloan Kettering Cancer Center. All slides of each case were re-reviewed, and the best representative formalin-fixed paraffin-embedded tumor block was chosen for construction of tissue microarray (TMA). A TMA was created using three 0.6 mm diameter punches per tumor. Thirty cores of normal pancreatic tissue were included as controls.

### Immunohistochemistry

TMA sections were immunolabeled, using the standard avidin-biotin peroxidase method, with antibodies against calretinin (SP65, Ventana) as well as two other mesothelioma markers, D2-40 (Signet) and WT-1 (WT49, Leica). For calretinin and D2-40, labeling was cytoplasmic, and for WT-1, labeling was nuclear. For all antibodies, labeling in at least 10% of cells was regarded as expression (labeling in 10-25% of cells was regarded as focal).

### Statistical Analysis

Mean, standard deviation, median and ranges were used to describe quantitative variables. Kaplan-Meier survival curves and the log-rank test were used for survival analysis. The Mann-Whitney u test or Fisher`s exact test was used to evaluate the differences in clinicopathologic features between Calretinin positive and Calretinin negative cases. *P*-values of <0.05 were considered to indicate statistical significance.

## RESULTS

### Clinicopathologic Findings

A total of 113 cases were included. Nine (8%) PDACs were labeled with calretinin. The mean age of the patients who were calretinin positive PDAC was 66.6 years. Six (67%) patients were female and three (33%) were male. Presenting symptoms included abdominal pain, nausea, vomiting, and weight loss. Three (33%) patients had jaundice and two (22%) had diabetes mellitus. One (11%) patient reported a family history of pancreas cancer. All patients were treated primarily by surgical resection (eight (89%) with pancreaticoduodenectomy, one (11%) with distal pancreatectomy); none received neoadjuvant chemotherapy.

Grossly, the tumors were mostly (89%) located in the head of the pancreas and the tumor size ranged from 1.5 cm to 4.2 cm (median, 3 cm). Six (67%) tumors had both lymphovascular and perineural invasion, and seven (78%) revealed metastasis in the lymph node(s). Only one (11%) tumor had a positive surgical margin.

When the calretinin positive cases and calretinin negative cases were compared, only the female:male ratio was found to be higher (2:1 vs. 1:1) in the former (p=0.49). The mean age was similar (66.6 vs. 67.8 years) (p=0.62), the tumors were mostly located in the head of the pancreas, and the median tumor size was the same (3 cm) (p=0.54) in both groups. Lymph node (78% vs. 69%) (p=0.71) and distant metastasis rates (33% vs. 34%) were also similar. Clinical and pathological characteristics of the cases are summarized in Table 1.

**Table 1:** Comparison of clinical and pathological features of calretinin positive and negative cases.

	**Calretinin positive** **(n=9)**	**Calretinin negative** **(n=104)**	**p value**
**Age years (mean ± SD)**	66.6 **± **10.2	67.8 ± 10.5	0.62*
**Female/Male**	6/3	52/52	0.49**
**Tumor location, n (%) **			
Head	8 (89)	75 (72)	
Body	0 (0)	4 (4)	0.68**
Tail	1 (11)	24 (23)	
Unknown	0 (0)	1 (1)	
**Median tumor size, cm (range)**	3 (1.5-4.2)	3 (1.3-9.8)	0.54*
**Tumor differentiation, n (%) **			
Well differentiated	0 (0)	3 (3)	
Moderately differentiated	4 (44)	79 (76)	0.03**
Poorly/Undifferentiated	5 (56)	22 (21)	
**Resection margin, n (%) **			
R0	8 (89)	95 (92)	
R1	1 (11)	8 (7)	0.54**
Unknown	0	1 (1)	
**Lymph node status, n (%) **			
N0	2 (22)	32 (30)	
N1	7 (78)	71 (69)	0.71**
Unknown	0 (0)	1 (1)	

*: Mann-Whitney u test, **: Fisher`s exact test, SD: Standard deviation.

### Immunohistochemical Findings

**Figure 1:**
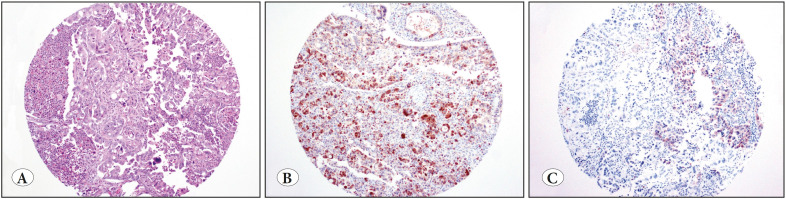
**A)** Pancreatic ductal adenocarcinoma (PDAC), poorly differentiated (H&E; x100). **B)** In our series, three poorly differentiated PDACs were diffusely and strongly positive for calretinin (IHC; x100). **C)** Two were focally and weakly positive for calretinin (IHC; x100).

**Figure 2:**
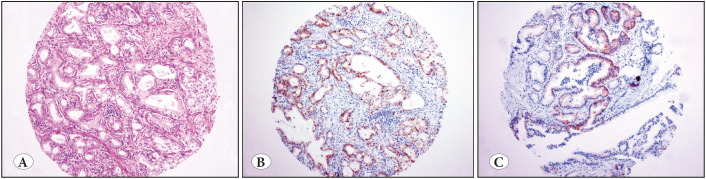
**A)** Pancreatic ductal adenocarcinoma (PDAC), moderately differentiated (H&E; x100). **B)** Three moderately differentiated PDACs were diffusely and strongly positive for calretinin (IHC; x100). **C)** One additional case was focally positive for calretinin (IHC; x100).

Five (56%) of these nine PDACs were poorly differentiated/undifferentiated carcinomas (Figure 1); three (60%) revealed diffuse and strong staining (Figure 1); two (40%) revealed focal and/or weak staining (Figure 1). The remaining four PDACs (44%) were moderately differentiated carcinomas (Figure 2); three (75%) revealed diffuse and strong staining (Figure 2), one (25%) revealed focal and/or weak staining (Figure 2). The incidence of calretinin expression was 15% in the poorly differentiated/undifferentiated carcinomas versus 6% in moderately differentiated carcinomas (p=0.03).

**Figure 3:**
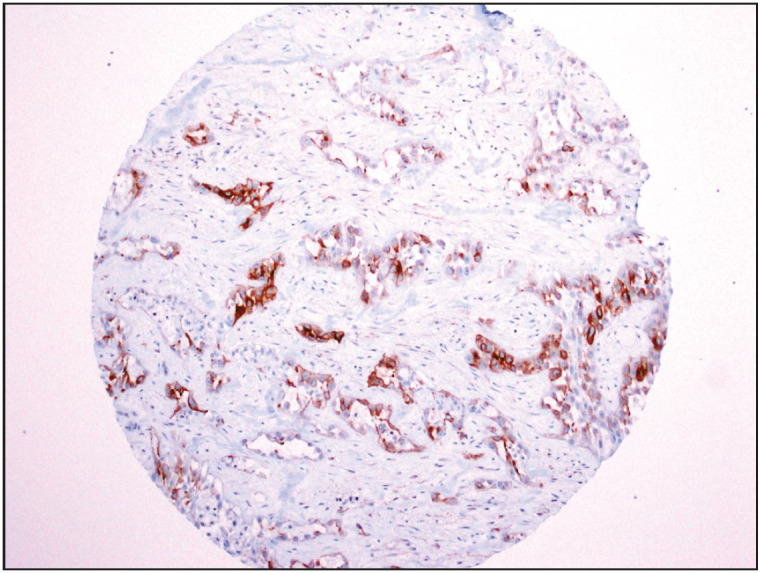
Only one pancreatic ductal adenocarcinoma expressed D2-40 (IHC; x100); this tumor was negative for calretinin or WT-1.

Only one PDAC, which was negative for calretinin, expressed D2-40 (Figure 3). There was no WT-1 expression in any of the 113 PDACs. Results of the immunohistochemical studies are summarized in Table 2.

**Table 2:** Results of the immunohistochemical studies.

**Antibody**	**Positive (%)**	**Negative (%)**
**Calretinin** Diffuse Focal	**9 (8)** 6 (67) 3 (33)	104 (92)
**D2-40**	**1 (1)**	112 (99)
**WT-1**	0 (0)	113 (100)

**Figure 4:**
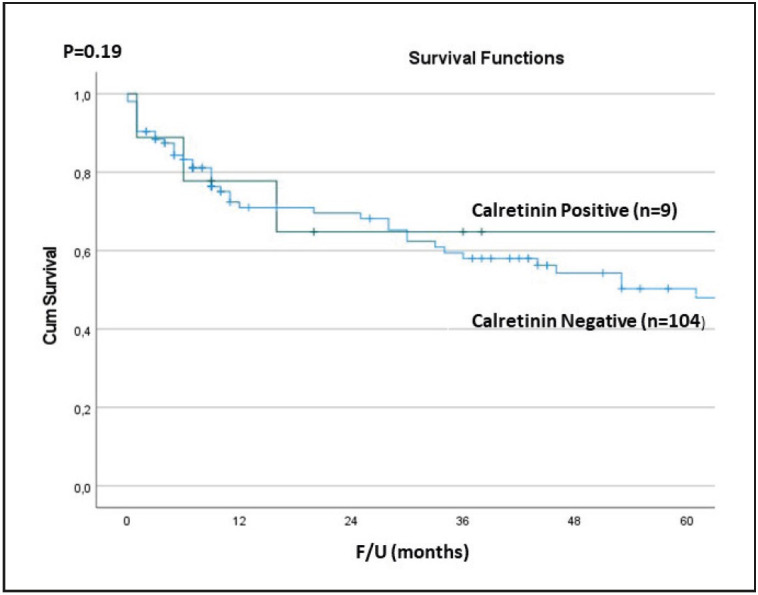
Survival outcomes of calretinin positive and calretinin negative cases.

**Outcome: **Clinical follow-up was available for all cases; the median follow-up was 16 months for the entire cohort (range, 1-143 months), 20 months for calretinin positive cases, and 12 months for calretinin negative cases. Of the nine calretinin positive cases, five (56%) died of the disease; one (20%) had local recurrence after 35 months; two (40%) had liver metastasis and one (40%) had peritoneal metastasis after 6, 9, and 54 months respectively. The remaining case (20%) had no local recurrence or distant metastasis. Four (44%) patients were alive with no evidence of disease, with a median follow-up of 11 months. There was no statistically significant difference between overall survival of calretinin positive cases and calretinin negative cases (p=0.19, Figure 4).

## DISCUSSION

Peritoneal involvement at presentation can be found in 40% of patients with advanced stage gastric carcinoma and almost 15% of patients with colorectal carcinoma (2,9,15,17–22). Similarly, pancreatic ductal adenocarcinoma is one of the most common causes of peritoneal carcinomatosis (1,3,23–25). Unfortunately, morphologic features are not enough to distinguish adenocarcinomas from peritoneal malignant mesothelioma in many cases as there are overlaps (26–29). Both tumor types may reveal polygonal, oval or cuboidal cells, with various grades of nuclear atypia and mitotic activity, arranged in tubulopapillary, micropapillary, solid, or even trabecular patterns. Moreover, intracytoplasmic mucin, a morphologic finding that would strongly favor adenocarcinoma, is usually not present in such cases as most of the adenocarcinomas are already poorly differentiated or undifferentiated at that stage. Therefore, a panel of immunohistochemical stains, including but not limited to calretinin, D2-40 and WT-1, is performed to establish the diagnosis because there is no single antibody sensitive and specific enough to prove (or argue against) mesothelial origin on its own (22,26,27,30–32).

For example, while sensitive for mesothelioma, calretinin expression has also been observed in a wide variety of poorly differentiated adenocarcinomas (12,16,33–35). Cargnello et al. reported that calretinin, while negative in all normal and adenomatous colorectal tissues, was expressed in 5-10% of colorectal adenocarcinomas and most of these cases were Grade 3 (i.e. poorly differentiated/undifferentiated) (10). Similarly, Liu et al. studied 257 colorectal adenocarcinomas (CRCs) and demonstrated calretinin positivity in three cases (1%). All three cases were poorly differentiated and revealed medullary features (36). There is no systematic study evaluating calretinin expression in PDACs.

In the current study, we analysed a large series of PDACs (n=113) and found that 8% of all PDACs express calretinin. When the calretinin positive cases and calretinin negative cases were compared, there were no significant differences: although calretinin expression was more common in females (F:M=2:1); the mean age, tumor location, the median tumor size, and the rates of lymph node and distant metastases were similar. Moreover, there was no statistically significant difference between the overall survival of calretinin positive and negative cases (p=0.19, Figure 4).

However, just like the calretinin positive colorectal adenocarcinomas, most (56%) of the calretinin positive PDACs were poorly differentiated or undifferentiated. Moreover, the incidence of calretinin expression was higher in the poorly differentiated/undifferentiated carcinomas compared to well/moderately differentiated carcinomas (15% vs. 6%, p=0.03). These observations show that when we really need help to distinguish an adenocarcinoma from peritoneal malignant mesothelioma, calretinin immunohistochemical stain may be misleading. Awareness of this phenomenon helps avoiding misinterpretations and prompts additional work-up leading to accurate tumor classification.

As mentioned above, D2-40 and WT-1 are the other markers that have been recommended frequently (27,31,32). D2-40, first described in glomerular epithelial cells, and then in lymphovascular endothelium, has been reported to reveal strong expression in up to 96% of malignant mesotheliomas, while it reveals only weak or no expression in adenocarcinomas (21,30,31,37–39). WT-1, originally discovered as a diagnostic marker for Wilms` tumor, is less sensitive than calretinin and D2-40 for peritoneal malignant mesothelioma but is more specific in distinguishing malignant mesothelioma from adenocarcinomas. In our study, only one tumor expressed D2-40, but this tumor was negative for calretinin. None of the PDACs were labeled with WT-1.

In conclusion, PDACs can be diffusely and strongly positive for calretinin creating a diagnostic pitfall for peritoneal metastasis, especially when the tumor is poorly differentiated or undifferentiated. Therefore, tumors expressing calretinin without other mesothelial markers such as D2-40 and WT-1 should prompt a careful evaluation of the morphologic and immunohistochemical features to exclude other peritoneal malignancies. If the diagnosis of PDAC is considered, ductal differentiation can be demonstrated by the combination of additional immunohistochemical markers such as mucin-related glycoproteins (MUC1 and MUC5AC) and/or oncoproteins (CEA, B72.3, etc.). 

## Conflict of INTEREST

The authors declare no conflict of interest.
